# Notice.—Elements of Human Anatomy

**Published:** 1854-01

**Authors:** 


ELEMENTS OF HUMAN ANATOMY—GENERAL, DESCRIPTIVE AND PRACTICAL. BY T. G. RICHARDSON, M. D., *Demonstrator of Anatomy in the Medical Department of the University of Louisville, and one of the attending Surgeons to the Louisville Marine Hospital.* Philadelphia—Lippincott, Grambo & Co., 1854. 8 vo., pp. 734.

A work on Anatomy by the accomplished Demonstrator in the University of Louisville, will be cordially welcomed by his numerous pupils and friends, not alone from feelings of personal interest, but because they know his ability to prepare an acceptable and useful treatise on the science to which he has been so long and zealously devoted. We feel safe in the prediction that the volume just issued will not fall short of their expectations.

We do not profess to have given the volume a thorough and critical perusal, but from what examination we have bestowed upon it, and guided by the opinions of those much more compe-

---

tent than ourselves to judge of the merits of a publication of this character, we believe that it will prove creditable alike to the author, as an accurate student of anatomy, and a clear, systematic instructor in that important province of medical education, to the University with which he is connected, and to our native medical literature. We do not doubt that, in proportion as its merits are ascertained, it will be adopted as an elementary text book in the different medical institutions of our country, and thus obtain a wide-spread circulation.

The plan and scope of the work are enunciated in the following quotation from the preface:—"In adding another to the numerous list of books on anatomy already before the profession, the author claims no credit beyond that of a common observer, whose experience, in the dissecting-room and amphitheatre, has suggested what he believes to be a slight improvement on the plan pursued by the majority of writers on this branch of physico-medical science. This improvement consists, in the first place, in the union of general, descriptive, and practical anatomy in the same volume; the intention being to render it unnecessary, on the part of the student just entering upon the study, to provide himself with separate books on these different branches; secondly, in the arrangement of the section devoted to practical anatomy so as to secure the greatest possible economy of material; and, lastly, in the substitution of English for Latin terms, wherever this appeared to be practicable and judicious."

In mechanical execution the work is excellent. The subjects are illustrated by numerous engravings in wood, which are unusually fine in distinctness of representation. We congratulate the author on this his first bibliographical achievement, trusting that hereafter similar occasions for congratulation may be often repeated. The work is for sale by Maxwell & Co.

